# Unlocking the Gut-Cardiac Axis: A Paradigm Shift in Cardiovascular Health

**DOI:** 10.7759/cureus.51039

**Published:** 2023-12-24

**Authors:** Akshay Akshay, Rayan Gasim, Thowaiba E Ali, Yash Sailesh Kumar, Ahmad Hassan

**Affiliations:** 1 Acute Medicine, Russell Hall Hospital, Dudley, GBR; 2 Internal Medicine, University of Khartoum, Khartoum, SDN; 3 Medicine and Surgery, University of Tennessee, Chattanooga, USA; 4 Medicine, Tbilisi State Medical University, Tbilisi, GEO; 5 Internal Medicine, Mayo Hospital, Lahore, PAK

**Keywords:** nutrition, diet, metabolites, immune responses, inflammation, heart-gut connection, microbial influence, gut microbiota, cardiovascular health, gut-cardiac axis

## Abstract

The gut-cardiac axis represents an emerging area of research focusing on the relationship between gut health and cardiovascular function. This narrative review examines the Gut-Cardiac Axis, emphasizing its emerging role in cardiovascular health and disease management.

Traditionally viewed as a component of the digestive system, the gut is now recognized for its significant influence on cardiac health. The gut microbiota, its metabolites, and gut-related inflammation are key factors affecting heart structure and function. This review highlights how dietary and nutritional interventions can effectively modulate the gut-cardiac axis, leading to personalized strategies for optimizing cardiovascular health. We discuss the clinical relevance of the gut-cardiac axis, particularly its role in providing diagnostic and prognostic markers for cardiovascular diseases.

This exploration of the gut-cardiac axis marks a significant shift in cardiology, integrating gut health into cardiovascular risk assessment and treatment strategies. The review provides an in-depth overview of current research and its potential to impact cardiovascular medicine significantly. We emphasize the importance of this research in advancing patient care and improving cardiac outcomes, underlining the potential of the gut-cardiac axis to transform cardiovascular health management.

## Introduction and background

The human body operates through a complex network of interconnected systems, each crucial for maintaining overall health and well-being. Medical research has shed light on the significant connections between various physiological processes in recent years. Among these, the evolving field of the "gut-cardiac axis" has emerged, demonstrating profound implications for cardiovascular health [1].

The term "gut-cardiac axis" encapsulates the dynamic relationship between the gastrointestinal system and cardiovascular health. This area of study, grounded in historical research, has become pivotal in understanding heart health. The early work of Ivan Pavlov in the 20th century, particularly his research on the cephalic phase of digestion, initially highlighted the brain-gut interactions. Pavlov's discoveries laid the groundwork for understanding interconnected body systems, including the heart-gut interaction [2]. Today's research delves deeper into this connection, revealing how the gut microbiome, inflammation, and metabolic pathways are intricately linked to cardiovascular well-being [3].

This review aims to explore the gut-cardiac axis in-depth, examining the latest research, clinical findings, and emerging paradigms. By traversing the fields of cardiology, gastroenterology, immunology, and endocrinology, we aim to unravel the complex interplay between the gut microbiota and cardiac function and the intertwined web of inflammation and immune responses that connect these two systems [4].

The gut-cardiac axis is not just a topic of academic interest; it holds significant potential to transform therapeutic approaches and prevention strategies for cardiovascular diseases. This field opens new avenues for personalized medicine, where treatments are tailored to an individual's unique microbiome and metabolic profile. Such an approach promises to enhance patient outcomes and propel the advancement of cardiovascular care [5].

## Review

Search strategy

An extensive search strategy was implemented to systematically explore the gut-cardiac axis and its impact on cardiovascular health. Our investigation focused on key concepts such as "gut-cardiac axis," "gut microbiota," and "cardiovascular health," along with associated metabolic, inflammatory, and immune aspects. Boolean operators were used to refine the search, combining phrases like "gut-cardiac axis AND cardiovascular health" and employing specific term searches including "short-chain fatty acids" (SCFAs) and "trimethylamine N-oxide" (TMAO).

The search strategy also incorporated truncation and wildcards to cover variations in terminology, ensuring a comprehensive coverage of the topic. Synonyms and related expressions were included to expand the research scope. Major databases like PubMed, Scopus, Web of Science, and Google Scholar were thoroughly searched. In addition, key journals such as the Journal of Cardiology, Gut, Nature Reviews Cardiology, and Circulation were reviewed. Filters were applied to select articles based on criteria like publication date, article type, and subject area.

To enhance our understanding and stay current with the latest developments, the references of relevant articles were examined, and citation tracking was employed. This search strategy was dynamic, adapting to the changing nature of our findings and the evolving field of gut-cardiac research. This approach ensured a comprehensive and updated review of the subject matter.

Mechanisms of the gut-cardiac axis

Gut-Derived Metabolites and Heart Health

The influence of gut-derived metabolites on the gut-cardiac axis is profound, particularly with SCFAs like butyrate and TMAO. SCFAs, generated through bacterial fermentation of dietary fibers, play a pivotal role in cardiovascular health. They regulate blood pressure and lipid metabolism and are crucial in mitigating cardiac ischemic injury [4]. Specifically, butyrate has been identified for its anti-inflammatory properties and is associated with decreased atherosclerosis risk [5].

Conversely, TMAO, produced from gut microbial metabolism of choline and carnitine, is linked to increased cardiovascular disease risks. High TMAO levels are associated with thrombosis, atherosclerosis, and myocardial infarction, underlining its detrimental impact on heart health [6].

Molecular and Cellular Pathways

The gut microbiota significantly influences cardiac health through immune-mediated mechanisms, metabolic byproducts, and direct cellular interactions. These microbial metabolites function as signaling molecules, influencing gene expression associated with inflammation, oxidative stress, and lipid metabolism in cardiac tissues [1].

Gut dysbiosis, or an imbalance in gut microbiota, can trigger systemic immune responses. This results in elevated pro-inflammatory cytokines, negatively impacting cardiac function. Such systemic inflammation is a critical factor in endothelial dysfunction, atherosclerotic plaque formation, and heart failure exacerbation [7]. The gut-cardiac axis also involves the autonomic nervous system, notably the vagus nerve, which modulates heart rate and inflammatory responses. Research indicates that variations in gut microbiota composition can affect cardiac autonomic function, thereby influencing heart rate variability and the risk of arrhythmias [8].

**Figure 1:**
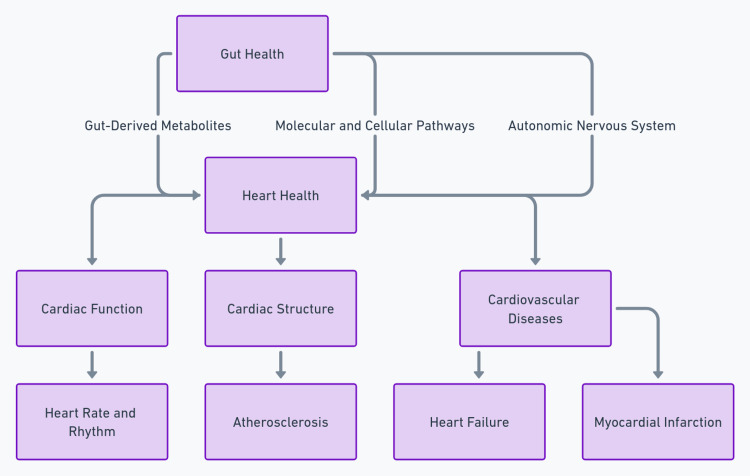
Exploring the gut-cardiac axis: a visual guide to interconnected health The image is generated by the authors.

These insights into the gut-cardiac axis highlight the intricate connections between gut-derived metabolites and molecular and cellular pathways in the heart. Understanding these mechanisms is essential for developing targeted interventions to lower cardiovascular risks and enhance overall heart health. A summary of the whole discussion is illustrated in Figure 1.

Clinical evidence and case studies

Recent Clinical Trials

Current clinical trials have demonstrated the practical implications of the gut-cardiac axis. A notable study by Tang et al. explored the association between TMAO levels and cardiovascular risk [9]. The study established a significant correlation, indicating that higher plasma levels of TMAO are linked to an increased risk of major adverse cardiovascular events. This finding emphasizes the critical impact of gut-derived metabolites such as TMAO on cardiovascular health.

Further contributing to this research area, Schiattarella et al. examined the effects of gut microbiota alterations in patients with heart failure [10]. The trial revealed distinct differences in gut microbiota composition between heart failure patients and healthy individuals, marked by a notable shift toward dysbiosis. This dysbiosis, characterized by decreased beneficial bacteria and increased pathogenic species, correlated with markers of cardiac dysfunction. These results underscore the direct and significant connection between gut microbiome health and heart failure, adding a vital perspective to our understanding of the gut-cardiac axis.

Case Study Highlights

Case studies provide valuable insights into the direct impact of changes in gut microbiota on cardiovascular health. One case, documented by Mayer et al., involved an individual with a history of atherosclerosis who underwent an analysis of their gut microbiota [8]. The study revealed significant dysbiosis, characterized by an abundance of bacteria known to produce TMAO. After implementing dietary changes to reduce TMAO-producing bacteria, the patient exhibited decreased TMAO levels and improved cardiovascular health indicators.

**Figure 2:**
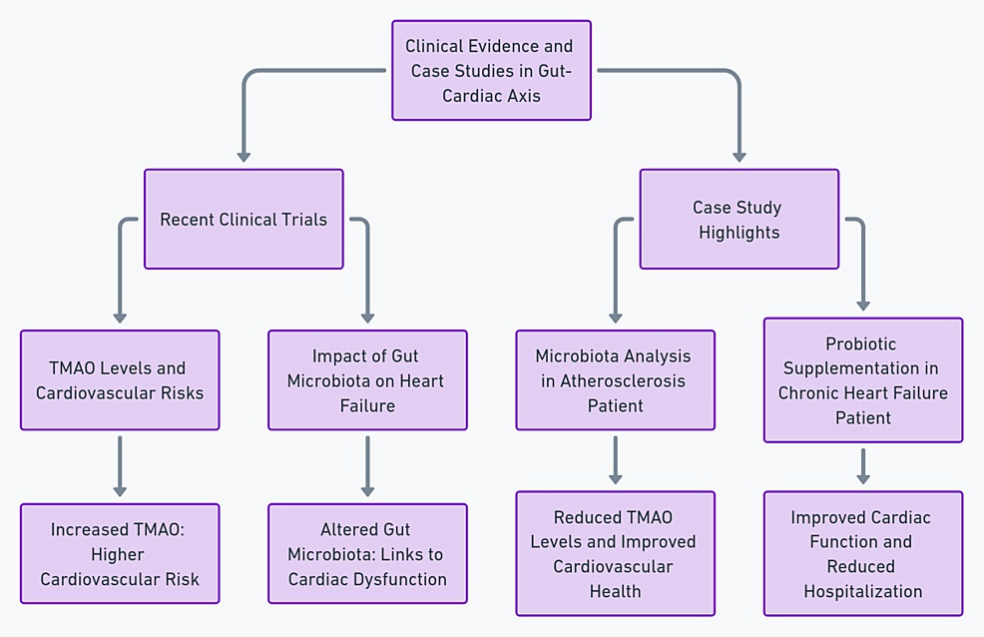
This diagram provides a structured overview of pivotal clinical evidence and case studies that have significantly advanced our understanding of the gut-cardiac axis The authors generated the image.

Another case reported by Jia et al. described a patient with chronic heart failure who received treatment supplemented with specific probiotics to modulate the gut microbiota [11]. This intervention led to improved cardiac function and a reduced frequency of hospitalization due to heart failure. This case highlights the potential efficacy of microbiota-targeted therapies in managing cardiac conditions. A summary of the whole discussion is illustrated in Figure 2.

Genetic and epigenetic influences

Genetic Predispositions

Genetic predispositions significantly influence gut microbiota composition, impacting cardiovascular health. Goodrich et al. found that specific genetic markers are linked to the presence and abundance of particular gut bacteria [12]. These genetic factors affecting the gut microbiome can predispose individuals to cardiovascular diseases. For instance, Khera et al. discovered that genetic variants associated with changes in the gut microbiota are also related to an increased risk of coronary artery disease. This evidence suggests that genetic predispositions may indirectly affect cardiovascular health via the gut microbiota [13].

Epigenetic Factors in Cardiovascular Health

Epigenetic factors, encompassing lifestyle and environmental influences, play a crucial role in modulating gene expression within the gut-cardiac axis. Factors such as diet, physical activity, and exposure to environmental pollutants can induce epigenetic changes impacting both heart and gut microbiome health. Research conducted by Kashtanova et al. demonstrated that dietary patterns could lead to epigenetic modifications influencing metabolic pathways relevant to cardiovascular diseases [14]. Additionally, chronic stress, known to alter gut microbiota composition, can result in epigenetic changes that elevate cardiovascular risk, as shown in studies by Kiecolt-Glaser et al. [15]. These findings underscore the significance of genetic and epigenetic factors in the comprehensive understanding and management of the gut-cardiac axis.

Establishing the connection

The Complex Interplay: Understanding the Gut-Cardiac Link

The intricate interplay between the gastrointestinal and cardiovascular systems defines the gut-cardiac axis. To fully understand this relationship, exploring the connections that link these two systems is crucial. Central to this connection is the gut microbiome. Recent studies have shown that the gut microbiota, far from being just a digestive aid, significantly regulates various aspects of cardiac function. It communicates with the heart through diverse pathways, including immune signaling, microbial metabolites, and neural connections [16].

The impact of the gut on cardiac health extends beyond its microbial inhabitants. The gut's role in the modulation of systemic inflammation and the immune system also affects cardiac health. Current research indicates that the integrity of the gut barrier is critical in balancing immune responses. A compromised gut barrier allows microbial products like lipopolysaccharides (LPS) to enter the bloodstream, triggering inflammation. This gut-derived inflammation can lead to endothelial dysfunction, atherosclerosis, and other cardiovascular diseases.

The influence of the gut on the immune system is profound, shaping cardiac health. Gut microbiota dysbiosis can activate the immune system, producing pro-inflammatory cytokines and other mediators. These can travel to the cardiovascular system, promoting inflammation and contributing to the development of various cardiovascular diseases. The role of monocytes and macrophages in atherosclerotic plaque development underscores the importance of understanding the gut-cardiac axis in the context of immunity.

Communication Pathways Between the Gut and Heart

The communication pathways between the gut and the heart are complex and multifunctional. Research highlights the role of gut-derived metabolites like SCFAs in cardiac function regulation [16]. These metabolites act as messengers in the gut-cardiac dialogue, influencing processes such as inflammation and oxidative stress in the heart.

Moreover, the connection between the gut and the heart extends to the autonomic nervous system. The "gut-brain-cardiac axis" field focuses on how the nervous system mediates communication between these organs [17]. The vagus nerve, for example, is critical in transmitting signals between the gut and the heart, influencing heart rate, rhythm, and arrhythmia risk.

Understanding these communication pathways sheds light on how the gut influences cardiac function. This knowledge is essential for deeper exploration and insights into the gut-cardiac axis.

Gut microbiome and heart health

Gut Microbiota: A Novel Player in Cardiovascular Health

The gut microbiota's role in cardiovascular health represents a significant shift in medical understanding. Previously seen mainly as a digestive component, the gut microbiome is now acknowledged as a critical player in cardiovascular health. Recent research highlights how the composition and diversity of gut microbes substantially affect cardiovascular outcomes [6].

This paradigm shift is based on the understanding that gut bacteria produce metabolites like SCFAs and TMAO. These metabolites significantly impact systemic inflammation, lipid metabolism, and endothelial function. They act as critical messengers and modulators in the gut-cardiac interaction, influencing the risk of atherosclerosis, hypertension, and other cardiovascular conditions [18].

The Microbial Impact on Cardiac Function

The influence of the gut microbiota on cardiac health goes beyond systemic effects to directly impact cardiac function. Research indicates that the gut microbial community can directly alter the heart's structure and functionality. Microbial products and metabolites can induce chronic low-grade inflammation in the heart, contributing to conditions like myocardial infarction and heart failure [19].

Recent studies have also explored the role of microbial regulation of the autonomic nervous system and its significant implications for cardiac health. This regulation affects heart rate and rhythm, potentially increasing the susceptibility to arrhythmias [9]. Dysbiosis in the gut microbiota, characterized by an imbalance in microbial communities, has been linked to changes in heart rate variability, a measure of the heart's adaptability. Such dysbiosis can disrupt the balance in the autonomic nervous system, affecting cardiac response to stressors and potentially leading to rhythm disturbances [5].

Understanding how the gut microbiota regulates the autonomic nervous system enhances our comprehension of the gut-cardiac axis. It highlights the complex interactions within the human body and opens new avenues for therapeutic interventions in cardiovascular medicine.

Inflammation, immunity, and cardiovascular disease

Inflammation in the Gut and Its Influence on Cardiac Inflammation

Inflammation is crucial in the gut-cardiac axis, significantly impacting cardiovascular health. Traditionally, inflammation within the cardiovascular system has been linked to conditions like atherosclerosis and heart disease. Recent research, however, has highlighted a direct link between gut inflammation and cardiac inflammation. This connection is vital as it demonstrates how the gut can modulate systemic inflammation, subsequently influencing the development and progression of cardiovascular diseases [20].

A key element in this relationship is the gut barrier. When this barrier is compromised, microbial products can enter the bloodstream, initiating an inflammatory response [21]. This gut-derived inflammation contributes to endothelial dysfunction and the formation of atherosclerotic plaques. Understanding the influence of gut inflammation on cardiac inflammation is essential for developing interventions to reduce cardiovascular risks [22].

Immune Responses Linking Gut Health to Cardiovascular Diseases

The immune system plays a central role in the gut-cardiac axis, bridging gut health and cardiovascular diseases. Studies have shown that an imbalance in gut microbiota, or dysbiosis, can activate the immune system, producing pro-inflammatory cytokines [3]. These cytokines can then travel to the cardiovascular system, promoting inflammation and contributing to the onset of cardiovascular diseases.

Furthermore, the impact of the gut on the immune system goes beyond systemic inflammation. It also affects the regulation and activity of immune cells like monocytes and macrophages, vital in developing atherosclerotic plaques. These immune responses directly link gut health and cardiovascular diseases, highlighting the importance of understanding the gut-cardiac axis in the context of immunity [23].

We can identify potential therapeutic targets by exploring the complex relationship between gut inflammation, immune responses, and cardiovascular diseases. These targets could help prevent or mitigate the effects of inflammation on heart health, paving the way for more effective treatments in cardiovascular medicine.

Metabolism at the crossroads

Metabolic Implications of the Gut-Cardiac Axis

The gut-cardiac axis represents a crucial intersection of metabolic processes significantly influencing cardiovascular health. Both systemic and microbial metabolism play vital roles in this complex relationship. Key metabolic aspects of the gut-cardiac axis include lipid metabolism, glucose homeostasis, and overall energy balance [24].

A central component of this interplay is the role of gut microbes in modulating metabolic processes. The gut microbiota is involved in transforming dietary components and endogenous molecules, leading to the production of various metabolites with significant effects on health. SCFAs, for instance, are metabolites produced by gut bacteria during the fermentation of dietary fibers. These SCFAs act as metabolic signaling molecules and are known to regulate lipid metabolism, inflammation, and energy homeostasis, all of which directly influence cardiac health [25].

Gut-Produced Metabolites and Their Role in Heart Health

Gut-produced metabolites are pivotal in shaping the gut-cardiac axis. These compounds, acting as biochemical messengers, significantly affect cardiovascular health. TMAO, a metabolite resulting from the microbial metabolism of dietary choline and carnitine, is a prime example. TMAO has received considerable attention due to its involvement in atherosclerosis, thrombosis, and other cardiovascular conditions [6].

The impact of these gut-derived metabolites on the cardiovascular system is diverse. They modulate inflammation, oxidative stress, blood pressure regulation, and endothelial function. Recognizing the role of these metabolites enhances our understanding of the metabolic communication between the gut and the heart.

Exploring the metabolic implications of the gut-cardiac axis provides insights into how metabolic processes affect cardiac health. This understanding is crucial for developing targeted interventions and therapeutic strategies to improve cardiovascular health.

Dietary influences and nutritional strategies

The Power of Diet: Nourishing the Gut and Heart

Diet plays a critical role in shaping the gut-cardiac axis, offering a means to nourish the gut and the heart simultaneously. The efficacy of diet in this context lies in its ability to alter the composition and function of the gut microbiota, thereby impacting cardiovascular health [26]. Diets enriched with dietary fibers, prebiotics, and probiotics can promote diverse and balanced gut microbiota, enhancing gut health and positively influencing cardiac outcomes [27].

Dietary choices also directly affect the production of gut-derived metabolites. For instance, foods high in choline and carnitine contribute to increased TMAO levels, a metabolite associated with heightened cardiovascular risk and atherosclerosis. Recognizing the dietary elements that either support or undermine the gut-cardiac axis is essential for developing effective heart-healthy nutritional strategies [28].

Nutritional Approaches for Cardiovascular and Gut Well-Being

Nutritional strategies within the gut-cardiac axis framework present a promising route for bolstering cardiovascular and gut well-being. These strategies encompass dietary patterns to optimize gut health, reduce inflammation, and address metabolic risk factors linked to heart disease [29].

Key nutritional strategies include adopting diets abundant in antioxidants, polyphenols, and omega-3 fatty acids, known for their cardioprotective properties. Furthermore, precision nutrition, which involves tailoring dietary plans to an individual's specific gut microbiota profile, can enhance the gut-cardiac axis and aid in preventing cardiovascular diseases. Adjusting dietary fiber intake is also crucial, as fibers act as substrates for the gut microbiota, producing beneficial metabolites such as SCFAs [30].

This exploration into the role of diet underscores its significant influence on gut and heart health. It goes beyond standard dietary recommendations, highlighting the fundamental role of dietary choices in shaping the gut's microbial composition, producing bioactive metabolites, and modulating systemic inflammation. Embracing this holistic approach acknowledges the comprehensive impact of diet on our health, emphasizing its role as a mediator between the gut microbiota and the heart.

Clinical significance

The Gut-Cardiac Axis in Disease: Implications for Clinical Practice

The gut-cardiac axis transcends theoretical understanding, representing a dynamic interplay between interconnected systems with significant implications for clinical practice. In the evolving healthcare field, comprehending this relationship's clinical relevance is crucial for healthcare professionals and researchers. This understanding aids in applying theoretical knowledge to practical settings. The gut-cardiac axis is fundamental in addressing cardiovascular diseases, influencing critical aspects of clinical care such as diagnosis, risk assessment, and therapeutic strategy development.

Recognizing the gut-cardiac axis as a critical factor in cardiovascular health marks a paradigm shift in clinical cardiology. This connection is associated with various cardiovascular diseases, including atherosclerosis and heart failure. The composition of gut microbiota, their metabolites, and related immune responses are increasingly recognized as valuable for diagnostic and prognostic purposes, introducing new dimensions to cardiovascular risk assessment. This approach aligns with the movement toward personalized medicine, where treatments are tailored based on individual gut microbial profiles, thereby improving therapeutic outcomes [1].

Recent studies have elucidated the role of gut microbiota in developing cardiovascular diseases, such as atherosclerosis, heart failure, and hypertension. Dysbiosis, or imbalance in gut microbiota, can lead to systemic inflammation and the production of pro-inflammatory molecules, contributing to cardiac dysfunction [19]. The clinical assessment of gut microbiota composition and activity offers potential as a tool for cardiovascular risk stratification and disease management.

Therapeutic avenues and future possibilities

The clinical significance of the gut-cardiac axis extends beyond diagnostics to include the exploration of novel therapeutic avenues and future possibilities [31]. Modifying the gut microbiota through dietary interventions, prebiotics, probiotics, or fecal microbiota transplantation shows promise in reducing cardiovascular risk [30]. Personalized nutrition, which customizes nutritional plans based on an individual's specific gut microbiota profile, can revolutionize preventive and therapeutic strategies for cardiovascular diseases [27].

Moreover, the gut-cardiac axis presents opportunities for innovative drug development. Targeting gut-derived metabolites, such as TMAO, offers new pathways for pharmacological interventions to mitigate cardiovascular risk [6]. Developing therapeutic approaches that restore gut homeostasis and reduce gut inflammation is crucial for improving cardiac outcomes in patients with cardiovascular diseases [16].

As we continue to explore the gut-cardiac axis's clinical significance, we uncover its transformative potential in cardiovascular medicine. Recognizing the intricate relationship between the gut and the heart paves the way for innovative and personalized medical approaches. These advances hold the promise of revolutionizing clinical practice and enhancing patient care, providing new horizons in treating and managing cardiovascular diseases.

Emerging frontiers in research

Contemporary Trends in Heart-Gut Connection Research

Exploring the heart-gut connection is dynamic and evolving, with current trends significantly influencing the research landscape. Emerging frontiers offer new insights into the complex relationship between the gut and the heart, paving the way for innovative discoveries and applications [31].

A primary focus of recent research is the role of gut health and microbiota in cardiovascular disease. Therapies targeting the microbiota, such as probiotics, prebiotics, and specialized dietary interventions, are at the forefront of this research. These approaches aim to restore gut homeostasis, modulate the effects of gut-derived metabolites, and reduce systemic inflammation, thus presenting promising strategies for cardiovascular risk reduction [30].

Precision medicine is becoming increasingly relevant in heart-gut connection research. Personalized nutrition, tailored to individual gut microbiota profiles, genetic makeup, and lifestyle factors, is critical to this approach. It involves customizing dietary recommendations to promote a healthy gut microbiome and overall well-being. Microbiome-based therapies, including fecal microbiota transplantation and pharmacological treatments targeting specific microbial pathways, are also gaining attention. These personalized strategies are anticipated to become integral in cardiovascular care, aligning with the unique microbial profiles of patients [27].

Unanswered Questions and Future Directions

Despite significant advances, heart-gut connection research still faces unanswered questions and challenges. Determining the precise mechanisms of how the gut microbiota affects cardiac function and vice versa remains a complex task at the intersection of microbiology and cardiology. Future research aims to elucidate the specific roles of gut bacteria and their metabolites in cardiovascular health. This ongoing research journey seeks to reveal the subtle intricacies of the gut-cardiac axis and open new avenues for therapeutic advancements in cardiovascular medicine [3].

Future research will also explore the gut-brain-heart axis, delving into the neurobiological and psychological aspects of the gut-cardiac connection. Areas such as the role of the vagus nerve and the gut's influence on mood, appetite, and stress responses are promising fields of inquiry. Additionally, investigating how factors like sex and age affect the heart-gut connection is crucial and remains largely unexplored [17].

These emerging frontiers in heart-gut connection research offer exciting prospects. Addressing these unanswered questions and exploring new research avenues can fully unlock the gut-cardiac axis's potential, transforming cardiovascular health management and introducing innovative preventive and therapeutic interventions [32].

Limitations of the Current Review in the Context of Gut-Cardiac Axis Research

While providing a comprehensive exploration of the gut-cardiac axis, this narrative review has limitations. The rapidly evolving nature of this field means new research could modify some of the conclusions presented here. Our review might cover only relevant research on the gut-cardiac axis, potentially overlooking additional insights. Most discussed studies are observational, establishing associations but not causation. There is also a potential selection bias in the chosen studies. Moreover, the therapeutic and diagnostic applications discussed are still early and require further clinical validation. Recognizing these limitations is vital for a balanced understanding of the gut-cardiac axis's current research state and its role in cardiovascular health.

## Conclusions

The exploration of the gut-cardiac axis marks a pivotal advancement in cardiovascular health, revealing the critical interplay between the gut and the heart. This connection, once underappreciated, is now recognized for its significant impact on cardiac well-being. Influences from gut microbiota, metabolites, and inflammation are now understood to directly affect heart structure and function. This shift in understanding has led to the development of personalized dietary and nutritional interventions, reshaping the landscape of cardiology risk assessment and treatment.

This advancement in gut-cardiac axis research is not just an academic milestone; it signifies a transformative shift in medical practice and patient care. It paves the way for innovative treatment strategies and drug development, fostering interdisciplinary collaboration and offering new hope for enhanced cardiac outcomes. The gut-cardiac axis stands as a symbol of progress and potential in medicine, promising a future of improved health care and a deeper understanding of cardiovascular diseases.
